# Molecular modeling and lead design of substituted zanamivir derivatives as potent anti-influenza drugs

**DOI:** 10.1186/s12859-016-1374-1

**Published:** 2016-12-22

**Authors:** Dhwani Dholakia, Sukriti Goyal, Salma Jamal, Aditi Singh, Asmita Das, Abhinav Grover

**Affiliations:** 1 Department of Biotechnology, Delhi Technological University, New Delhi, 110042 India; 2 Department of Bioscience and Biotechnology, Banasthali University, Tonk, Rajasthan 304022 India; 3 Department of Biotechnology, TERI University, New Delhi, 110070 India; 4 School of Biotechnology, Jawaharlal Nehru University, New Delhi, 110067 India

**Keywords:** Neuraminidase, H1N1, H3N2, NA, Influenza, QSAR

## Abstract

**Background:**

Influenza virus spreads infection by two main surface glycoproteins, namely hemagglutinin (HA) and neuraminidase (NA). NA cleaves the sialic acid receptors eventually releasing newly formed virus particles which then invade new cells. Inhibition of NA could limit the replication of virus to one round which is insufficient to cause the disease.

**Results:**

An experimentally reported series of acylguanidine zanamivir derivatives was used to develop GQSAR model targeting NA in different strains of influenza virus, H1N1 and H3N2. A combinatorial library was developed and their inhibitory activities were predicted using the GQSAR model.

**Conclusion:**

The top leads were analyzed by docking which revealed the binding modes of these inhibitors in the active site of NA (150-loop). The top compound (AMA) was selected for carrying out molecular dynamics simulations for 15 ns which provided insights into the time dependent dynamics of the designed leads. AMA possessed a docking score of −8.26 Kcal/mol with H1N1 strain and −7.00 Kcal/mol with H3N2 strain. Ligand-bound complexes of both H1N1 and H3N2 were observed to be stable for 11 ns and 7 ns respectively. ADME descriptors were also calculated to study the pharmacokinetic properties of AMA which revealed its drug-like properties.

**Electronic supplementary material:**

The online version of this article (doi:10.1186/s12859-016-1374-1) contains supplementary material, which is available to authorized users.

## Background

Type A influenza virus, member of orthomyxoviridae family [1] is one of the most lethal and virulent strains of influenza virus which has been responsible for worldwide havoc including seasonal epidemics and major pandemic breakthroughs [2]. Pandemic Influenza could have originated via two major mechanisms, either by direct transmission from birds to humans as in 1918 “Spanish Influenza” virus (H1N1) or via genetic reassortment between avian influenza virus and human influenza virus as happened in the case of 1968 “Hong Kong” influenza virus (H3N2) [3]. It is a highly contagious virus and causes severe respiratory associated problems. Complications include post influenza encephalitis, secondary bacterial pneumonia and change in cardiac electrocardiogram [4, 5].

Subtypes of the type A virus has been classified based on the serological activity of the glycoprotein antigens hemagglutinin (HA) and neuraminidase (NA). Sixteen serotypes of HA have been found to circulate in mammalian and avian hosts. HA is a surface envelop protein of influenza virus and performs crucial viral functions like host recognition and membrane fusion [6]. HA often recognizes sialic acid receptors found in the human upper respiratory tract which act as initial key step of viral infection [7]. The second glycoprotein NA is a sialidase which destroys HA present on the surface of the virus allowing release of the infected viral progeny from infected cell thus preventing their self-aggregation [8]. Thus, inhibiting NA prevents second round of replication of influenza virus therefore culminating further influenza infection. Sequence analysis of nine subtypes of NA separates them into two major phylogenetic groups. Group 1 consists of N1, N4, N5 and N8 while group 2 consists of N2, N3, N6, N7 and N9.

Active site of NA is lined by 150-loop which includes residues from 147 to152 and is present in two forms. First is an open conformation which adopts 150-loop formation and the other is a closed conformation in which active site lacks 150-loop conformation [9]. Analysis of X-ray crystal structure [10] shows an open conformation for NA in group 1 and a closed conformation for those in group 2. However molecular dynamics simulation suggested the presence of 150-loop not only in group 1 but also in group 2 [9]. These findings provide deep insight into the design and synthesis of new NA inhibitors targeting the 150-loop lining cavity. Based on these structures FDA approved drugs [11] like Oseltamivir (Tamiflu), Zanamivir (Relenza) and Peramivir are commercially available to treat infected patients. Oseltamivir, an oral prodrug administered as phosphate, is hydrolysed hepatically to its active form carboxylate while Zanamivir is administered via nasal inhalation because of high polar compounds. To alleviate the potential consequence of suboptimal bioavailability and clumsy inhalational devices, an intravenous peramivir antiviral drug was used. However, this type of treatment has limited control as the developed vaccines frequently become ineffective because of mutation in influenza viral antigen taking place at a rapid rate in forms of antigenic shift or drift resulting in resistance [12]. Thus a search for new influenza drug with broad spectrum activity is the need of hour.

Considerable amount of work has been done to target the 150-loop lining cavity through modification of the existing inhibitors by attaching various additional groups with appropriate size, shape and hydrophobicity [2]. *In silico* methods provide substantial contribution to drug design and development of lead compounds in limited time and resources. Quantitative structure activity relationship (QSAR) is a method of ligand-based drug designing that establishes relationships between structure and inhibitory activity of inhibitors. Group-based QSAR (GQSAR) gives flexibility to traditional QSAR methods by calculating descriptors for the fragment of a molecule rather than calculating descriptors for whole molecule [13–16]. Unlike the traditional QSAR methods, GQSAR can be applied to both congeneric as well as non-congeneric series of compounds.

In this study we developed a novel GQSAR model based on congeneric series of acylguanidine zanamivir derivatives [17–19]. Same set of congeneric series were counter screened against NA of both H1N1 and H3N2. The main purpose of our study was to develop a robust GQSAR model to identify relation between structure and biological activity of the set of zanamivir derivatives as a function of fragments done at substitution site. Developed model predicted the relationship between anti-influenza activity and electro-chemical properties of the derivatives with high efficiency. Various descriptors essential for effective interaction between inhibitors and the active site of target were identified. An attempt has also been made to understand effect of different substituents at the substitution site in the template structure. In addition to building of GQSAR model, a comprehensive computational insights into binding action of lead compound to targets has also been provided.

## Methods

### Preparation and optimization of data set

Marvin sketch (ChemAxon Ltd., https://www.chemaxon.com/products/marvin/) was used to draw experimentally reported 24 acylguanidine zanamivir derivatives. The compounds were drawn in 2-D format and then converted to 3-D using VlifeEngine module of VLifeMDS [20]. The prepared compounds were minimized using force field batch minimization platform of VlifeEngine ver 4.3 provided by Vlife Sciences, Pune on Intel® Xeon(R).

### Calculation of descriptors for GQSAR model development

**Fig. 1:**
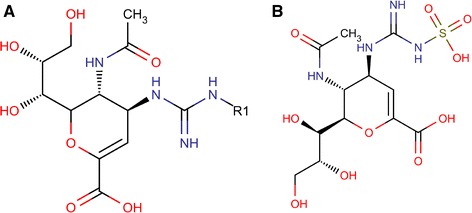
**a** Representation of common template for acylguanidine zanamivir derived compounds. **b** Designed novel lead compound AMA

In this GQSAR study, various descriptors correlating the inhibitory activity of molecules were identified as detailed in our previous publications [13–15]. GQSAR model was built using the GQSAR module of VlifeMDS [15]. The common scaffold, representative of all the structures was used as a template for the GQSAR study. Using Modify module of VLifeMDS, template (Fig. 1) was created by replacing dummy atoms at R1 on the common moiety i.e. template. Optimized set of compounds and template molecule were then imported for template based GQSAR model building. Experimentally reported IC50 values (half maximal inhibitory concentration) were converted to pIC50 scale (−log IC50) to narrow down the range (Additional file 1: Table S1). Thus, a higher value of pIC50 exhibits a more potent compound. These values were then manually incorporated in VLifeMDS. Physicochemical 2-D descriptors were calculated for functional group at substitution site (R1). Total of 101 descriptors out of 343 descriptors were further used for QSAR analysis while rest were removed owing to invariability.

### Development of GQSAR model using multiple regression method

For development of a robust and efficient model, the data set of compound was divided into training and test set. The data set was divided into training and test set by random distribution of 70% into training and remaining 30% into test set. For GQSAR against NA of H1N1, 16 molecules were grouped into training set while8 molecules namely f, l, n, o, q, t, y and Ae were grouped in test set. For the second NA target of H3N2, 16 molecules were chosen for training set and 8 molecules namely ac, ae, j, m, q, r, w, y were selected for test set. After division of training and test set, the unicolumn statistics for both the training and test sets were calculated which provides validation of the chosen training and test sets. Stepwise-forward method was used as variable selection. The next step involved, building of a GQSAR model using multiple regression analysis which predicts the activity using the selected descriptors. Regression analysis is process which estimates the relationship between a dependent variable and one or more independent variable. For this model Column containing the activity values (pIC50) was selected as dependent variable while rests other were selected as independent variables. In general, multiple regression can be explained in the terms of Eq. 1.

1 $$ Y=\alpha +{\beta}_0+{\beta}_1{X}_1+{\beta}_2{X}_2+\cdots +{\beta}_n{X}_n $$

Where Y is the independent variable, α is the intercept, β_n_ is the slope for n^th^ independent variable X.

### Validation and evaluation of the developed model

This step was done to test both the stability and predictive ability of the developed GQSAR models. Various statistical parameters [21] like k (number of variables), n (number of compounds), r^2^(Squared degree correlation), q^2^(cross validated correlation coefficient), Pred_r^2^(for external test set), Z score (Randomization test), F-Test, best_ran_q^2^ (Highest value of q^2^ in randomization test), best_ran_r^2^ (highest value of r^2^ in randomized test) and standard error were calculated to test goodness of fit of the developed model. For a model to be robust, values should be above threshold i.e. r^2^ > 0.6, q^2^ > 0.6, and Pred_r^2^ > 0.5 [21–23]. Higher values of F-Test and lower values of standard error of Pred_r^2^se, r^2^_se and q^2^_se indicate a statistically reliable model. Internal and external validation of the model was performed as detailed in our earlier publications [13–16].

### Development of combinatorial library

Combinatorial library was generated using the Leadgrow module of VLifeMDS by substituting various chemical groups at the substitution site R1 site. The library generated consisted of 189 molecules. Prediction of activity and descriptor for each of the substituted site was calculated using the developed GQSAR model via generic prediction module.

### Protein and ligand preparation for docking studies

The protein crystal structure of both H1N1 (PDB ID: 3BEQ) and H3N2 (PDB ID: 4GZ0) were retrieved from protein databank. Since the structures obtained were homomer complex structures, only the monomer chain was selected and rest including water and non-bonded atoms were removed using Accelyrs Viewer lite 5.0 [2, 15, 19]. The combinatorial library compounds with good predicted activity were selected and prepared using Ligprep and protein structure was prepared using Protein Preparation wizard [24–27].

### Receptor grid generation

A Glide scoring grid around the receptor was generated using receptor grid generation platform of Schrodinger’s Glide modules [28]. This utility of Glide defines receptor structure, determines and mark active site position. All the parameters were kept default and a grid of size 20 × 20 × 20 Å with inner box size of 10 × 10 × 10 Å was generated.

### Docking and scoring

The prepared combinatorial library compounds were docked against NA of H1N1 and H3N2 using extra precision GlideXP platform. The selected poses were further minimized on pre-computed OPLS-2005 electrostatic and van der Waals grid for receptor. Ultimately lowest energy poses were subjected to Monte Carlo minimization and rescored using Glide Score function. The complexes with least XP score (highest magnitude) were selected for molecular dynamics simulations.

### ADME prediction

Absorption, distribution, metabolism and elimination (ADME) of the selected compounds were predicted *in silico* using QikProp module of Schrödinger suite [29]. Ligands were initially prepared using LigPrep. It predicts physically significant descriptors and relevant pharmaceutical properties. In addition to the molecular descriptors, QikProp also provides their range values by comparing an individual molecule property with those known 95% drug.

### Molecular dynamics simulations

Docked complex of protein and ligand were prepared in protein preparation wizard of maestro. Desmond software was then used to study the molecular dynamics of ligand inside the active site of NA for both H1N1 and H3N2 using the Optimized potentials for liquid simulations 2005 (OLPS) force field [30]. Structures were uploaded in Desmond for further process of molecular dynamics simulations using parameters as mentioned in our earlier publications [16, 31]. The docked complexes were then simulated for 15 ns using above parameters. Frames of trajectory were recorded for each 10 ns time step. The root mean square deviations (RMSD) for the docked complexes were calculated for the entire simulations trajectory with reference to their respective frames. Radius of Gyration and hydrogen bond analysis were carried out for all the frames of 15 ns MD simulation.

## Results and discussion

### Separation of data into training and test set

A QSAR model was developed for acylguanidine zanamivir derivatives considering the activity and various physiochemical descriptors for both H1N1 and H3N2. Seventy percent of total compounds were selected as training set and the rest as test set. Separation of the dataset into training and test set was validated using unicolumn statistics (Tables 1 and 2) according to which maximum of test should be less than maximum of training set and minimum of test should be greater than minimum of training set [32].

**Table 1:** Unicolumn statistics for training and test sets for *influenza* H1N1 Neuraminidase inhibitory activity

Data set	Average	Max.	Min.	Std dev	Sum
Training	−2.4963	−1.3032	−4.5955	0.6975	−39.9406
Test	−2.5855	−1.7396	−4.5396	0.8352	−20.6838

**Table 2:** Unicolumn statistics for training and test sets for *influenza* H3N2 Neuraminidase inhibitory activity

Data set	Average	Max.	Min.	Std dev	Sum
Training	−2.5530	−1.7657	−4.4713	0.6407	−40.8485
Test	−2.5821	−1.4065	−4.5832	0.9057	−20.6564

### Analysis of GQSAR models developed against H1N1 and H3N2

A robust GQSAR model was developed which explained correlation between the physiochemical parameters and contribution of each substitution site. Several models were developed and the best model with significant values based on statistical parameters was chosen.

### H1N1 model

The chosen model for H1N1 exhibited significant statistical values of r^2^ (squared correlation coefficient) = 0.95, q^2^ (cross-validated squared correlation coefficient) = 0.90, Pred_r^2^ (predicted squared correlation coefficient) = 0.95, F-Test = 92.99 while standard errors were observed to be r^2^_se = 0.15, q^2^_se = 0.23, Pred_r^2^se = 0.18. Low standard error values indicated absolute quality of the model.

Three descriptors namely R1-SdOEindex, R1-6ChainCount and R1-SssSE-index were selected by the model for all the compounds. The model had good internal and external prediction. The model can be given by the Eq. 2.

2 $$ plC50=\left(23.61\kern0.62em \ast \kern0.62em R1-Sd 0 Eindex\right)+\left(47.12\kern0.62em \ast \kern0.62em R1-6 ChainCount\right)-\Big(39.90\kern0.62em \ast \kern0.62em R1-(SssSEindex)-5.26. $$

**Fig. 2:**
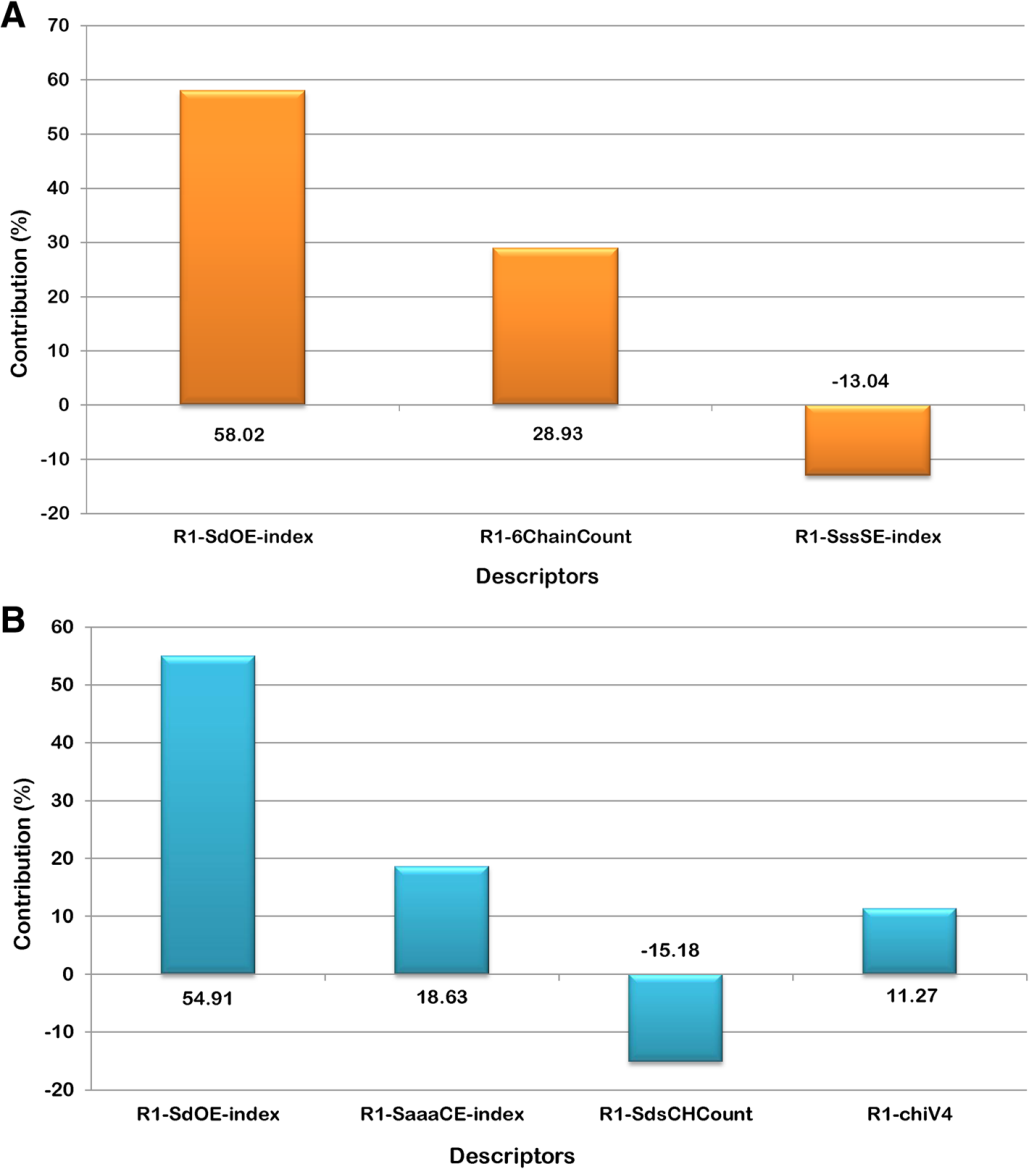
Contribution plot of GQSAR model developed against (**a**) H1N1 and (**b**) H3N2

**Fig. 3:**
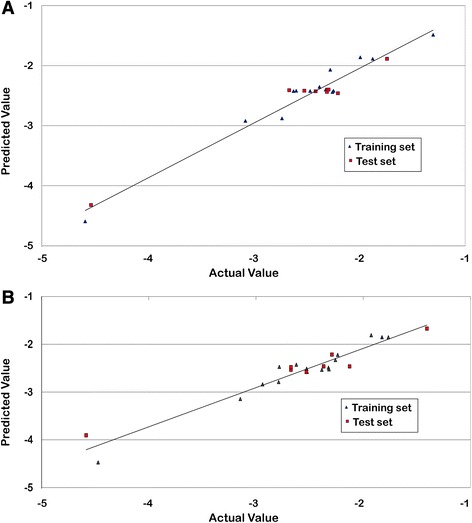
Graph of observed vs. predicted activity for training and test set of (**a**) H1N1 and (**b**) H3N2

**Fig. 4:**
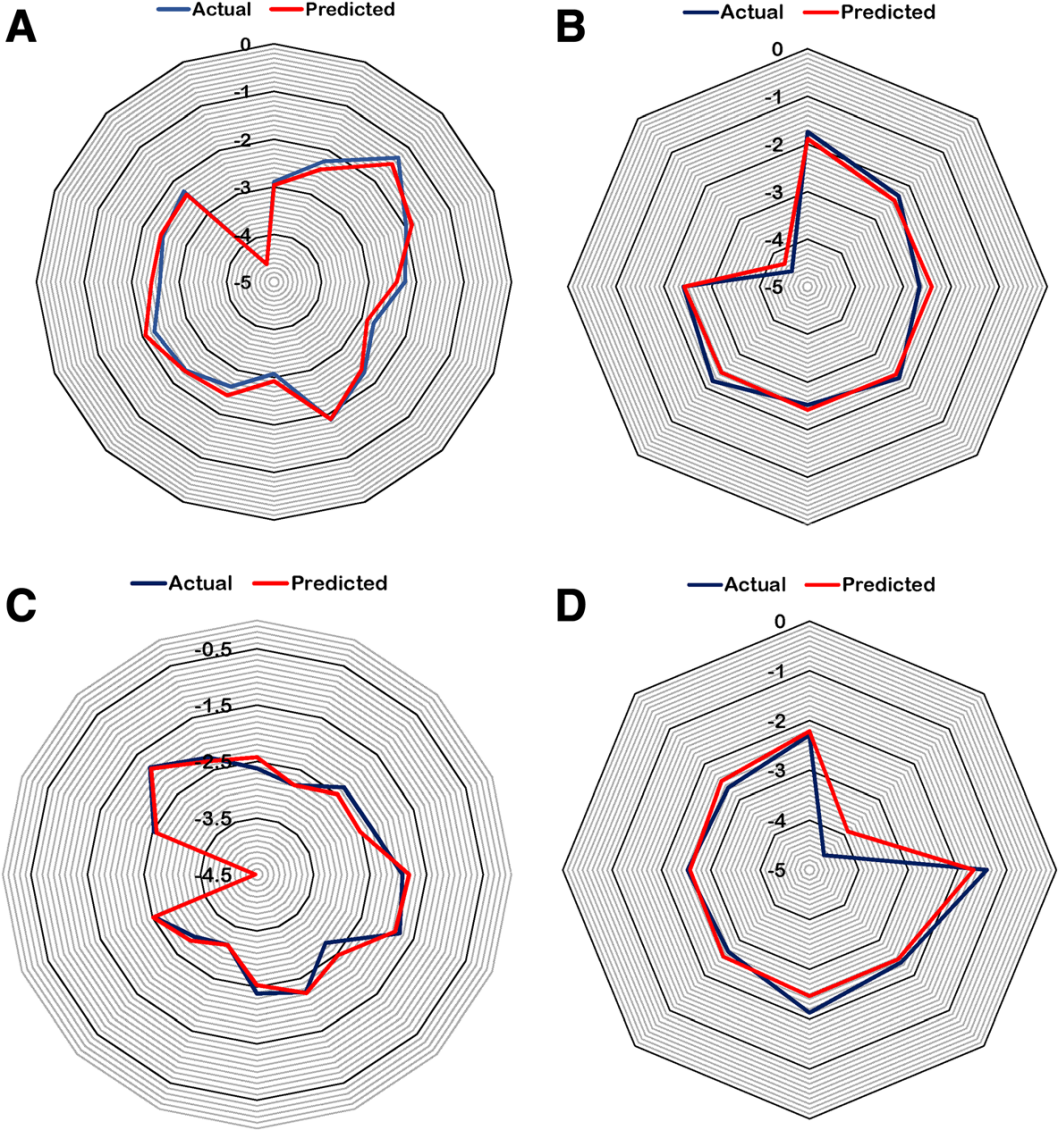
Radar plots showing observed and predicted values of (**a**) training set and (**b**) test set for H1N1 (**c**) Training set and (**d**) test set for H3N2

With n = 16, degree of freedom = 12, ZScore R^2^ = 3.35, ZScore Q^2^ = 0.69, “n” represents total number of compounds in the training set. The derived QSAR model shows a good correlation between aforementioned descriptors and biological activity as r^2^ is 0.95 with minimum standard error of 0.15. The p-value was observed to be < 0.001 for both models. The model incorporates various descriptors as shown in Table 3. R1-SdOEindex which is an electro-*topological* descriptor gives information about the number of –OH groups connected with one double bond. The positive contribution of 58.02% (Fig. 2a) indicates that presence of –OH group increases the inhibitory activity of the NA inhibitors. The percentage contribution is relative (not absolute) contribution of individual descriptors among the selected descriptors that are important for activity variation. These values are an indication of the relative importance of fragment-specific descriptors towards their contribution in the inhibitory activity of the ligands. Second descriptor, R1-6ChainCount is one of the most influential descriptors which signifies the total number of six-membered rings in a compound. Thus, a positive contribution of 28.93% indicates that the presence of aromatic compounds like phenyl could improve the inhibitory potency of compounds targeting NA. The third descriptor, R1-SssSEindex shows the importance of electronic environment of sulfur atom bonded with two single non-hydrogen atoms in the molecule. A negative contribution value of 13.04% suggests decrease in E-state contribution of either aromatic or free sulfur could improve the inhibitory activity. Thus, it can be deduced that the model is reliable and predictive, which can also be seen in the line graph of observed vs. predicted activity as shown in Fig. 3a and also the radar plots of observed and predicted activity for both training and test set (Fig. 4a and b).

**Table 3:** Physicochemical descriptors with predicted activity values for training and test set for H1N1 model

Column	R1-SdOE-index	R1-6ChainCount	R1-SssSE-index	Prediction
1186	17.51	0	0	−1.1278
1185	17.20	0	0	−1.2019
1189	13.03	2	0	−1.2442

### H3N2 model

The model developed against H3N2 also showed satisfactory statistical values with r^2^ = 0.95, q^2^ = 0.93, Pred_r^2^ = 0.87 and F-test = 61.02 and the standard errors as r^2^_se = 0.15, q^2^_se = 0.19, Pred_r^2^_se = 0.32. A line graph of observed vs. predicted activity is shown in Fig. 3b. Low standard error and high values of internal and external prediction indicate robustness of the model. Thus, it can be inferred that the model is reliable and predictive, which can also be seen in the radar plots of the observed and predicted activity for both training and test set (Fig. 4c and d). Four descriptors were selected for model namely R1-SdOEindex, R1-SaaaCEindex, R1-SdsCHcount, R1-chiV4. The developed model had a good internal as well as external prediction. The model can be explained via Eq. 3.

3 $$ plC50+\left(22.90\ast R1-Sd 0 Eindex\right)+\left(20.31\ast R1- SaaaC\underset{\_}{E} index\right)-\left(25.88\ast R1- SdsCHcount\right)+\left(26.58\ast R1- chiV4\right)-4.83 $$

with *n* = 16, degree of freedom = 11, ZScore R^2 = 5.94, ZScore Q^2 = 0.71, “n” represents total number of compounds in the training set.

The equation obtained above contains three physicochemical descriptors as shown in Table 4. Depending on the inhibitory activity of the data set compounds against H1N1 and H3N2, descriptors obtained for both the models were found to be different, indicating that the inhibitory activity of data set compounds is affected by different descriptors (as well as fragments) in the case of H1N1 and H3N2. R1-SdOEindex gives information about number of –OH groups connected with one double bond. The positive contribution of 54.91% (Fig. 2b) indicates that presence of –OH group at R1 position increases the inhibitory activity of the NA inhibitors. The second descriptor, R1-SaaaCEindex is an electro topological descriptor which indicates the number of carbon atoms that are connected with three aromatic bonds. A positive contribution (18.63%) indicates that increase in SaaaCE properties would enhance the inhibitory effect of lead compound. Another descriptor R1-SdsCHcount highlights the number of –CH groups connected with one double and one single bond in a molecule. Negative contribution of 15.18% indicates that increase in length of -CH atoms chain at the substitution site of NA inhibitors could be detrimental to the inhibitory activity. The last descriptor, R1-chiV4 is a steric property descriptor that helps in discriminating molecules according to size, degree of branching, shape and overall flexibility. A positive contribution of 11.27% indicates that increasing the steric properties at R1 will account for increased inhibitory activity.

**Table 4:** Physicochemical descriptors with predicted activity values for training and test set for H3N2 model

Column	R1-SdOE-index	R1-SaaaCE-index	R1-SdsCHcount	R1-chiV4	Prediction
1186	17.51	0	0	0	−0.823
1185	17.200	0	0	0	−0.894
1184	16.25	0	0	0	−1.112

### Combinatorial library analysis and selection of lead compound

Combinatorial library was generated after analyzing the above two models and inhibitory activities of the developed compounds were predicted. Various substituting groups like alkanes, atoms, aromatic rings, ketone, ester etc. were added. The developed library contained 189 molecules. Molecules having activity values more than that reported in congeneric series were selected and the compound having highest predicted activity was chosen as lead compound [2]. It was seen that lead compound (Fig. 1b) was substituted with sulphite group at R1 position and had good predicted activity value for H1N1 and H3N2. Docking studies were performed on lead compound and further molecular dynamics was also performed to check its stability in aqueous environment.

### ADME prediction

ADME properties were predicted using QikProp program (Schrodinger Inc). The IUPAC name of the lead compound docked is *(2R,3R,4S)-3-acetamido-4-{[(sulfoamino)methanimidoyl]amino}f-2-[(1R,2R)-1,2,3-trihydroxypropyl]-3,4-dihydro-2H-pyran-6-carboxylic acid* (AMA), details in the next section. It was found that AMA, highest scoring molecule followed three conditions of Lipinski rule of five. Various descriptors were evaluated for ADMET properties. The range values for each descriptor were given based on the known values of 95% of drugs. Molecular weight of AMA was found to be 412.4 (ideal molecular weight 130–725). Descriptors considered for drug permeability includes molecular volume of solute, hydrogen bond acceptor and liophilicity. Molecular volume of the compound was found to be 1107.4 (range value 500–2000) while hydrogen accepter was found to be 12.8 (range value 2–20). The latter parameter estimates average number of hydrogen bonds that would be accepted by the solute from water molecules in an aqueous solution. Rotatable bond count is one of the widely used descriptor that inversely correlates with oral bioavailability. Rotatable bonds of this compound had value of 12 (Range 0–15). Various computational parameters were also calculated to analyze the solubility from its 2-D structure. Solvent accessible surface area (SASA) is one influential parameter, which defines the surface area of biomolecule that can be accessed by solvent. It is usually performed using 14 Å radius which generates various components *viz.* total SASA whose value was found to be 618.8 (Range values 300–1000), solute hydrophobic SASA (FOSA) with value 154.1 (Range value 0–750), solute Carbon Pi SASA (PISA) whose value was found to be 25.7 (range value 0–450) and solute weekly polar SASA (WPSA) which includes surface area for all sulphur, halogens and phosphorous atom with a value of 2 (Range value 0–175). Distribution of lead compound in solution is calculated using the ionization potential parameter which affects the availability of the compound for further physical, chemical or biological reactions. The calculated descriptor Solute Ionization Potential (eV) was found to be 9.7 (Range value 7.9–10.5). Various other electrochemical descriptors like Solute Globularity (Sphere = 1) = 0.837 (0.75/0.95) and Solute Electron Affinity (eV) = 1.262 (−0.9/1.7) were also calculated. This lead compound was found to be similar to various compounds like Voglibose 68.40, Valganciclovir 68.04, Aminopterin 65.66, Lisinopril 64.63 and Methotrexate 64.39. All these above parameters suggest that AMA can be a potential drug molecule and a good lead candidate.

### Docking and molecular dynamics simulations studies of AMA with H1N1 and H3N2

**Fig. 5:**
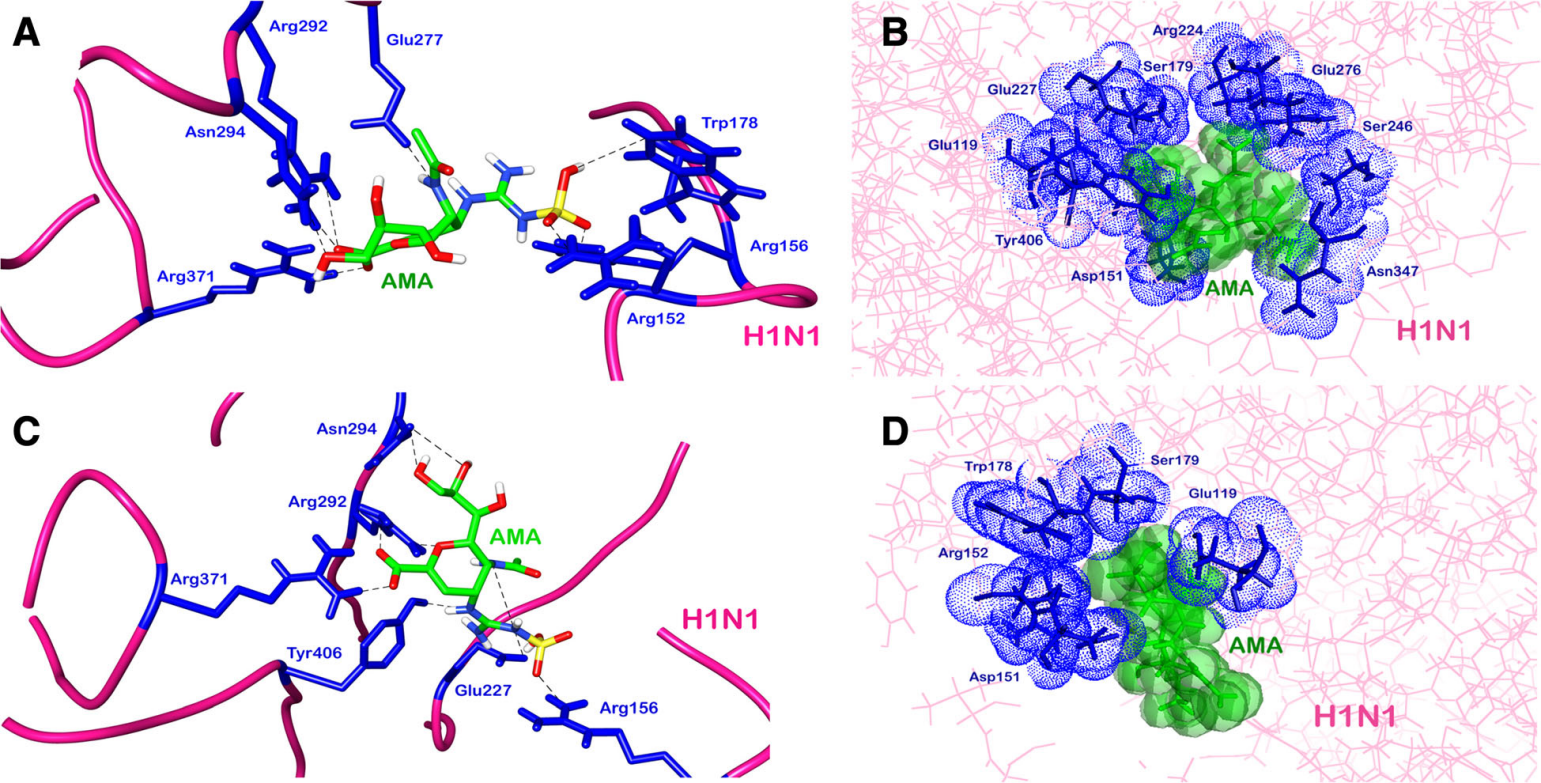
Molecular interactions of H1N1 Neuraminidase (*pink*) with AMA (*green*) depicting (**a**) hydrogen bond before MD simulations and (**b**) hydrophobic interactions before MD simulations. (**c**) Hydrogen bond after MD simulations and (**d**) hydrophobic interactions after MD simulations

**Fig. 6:**
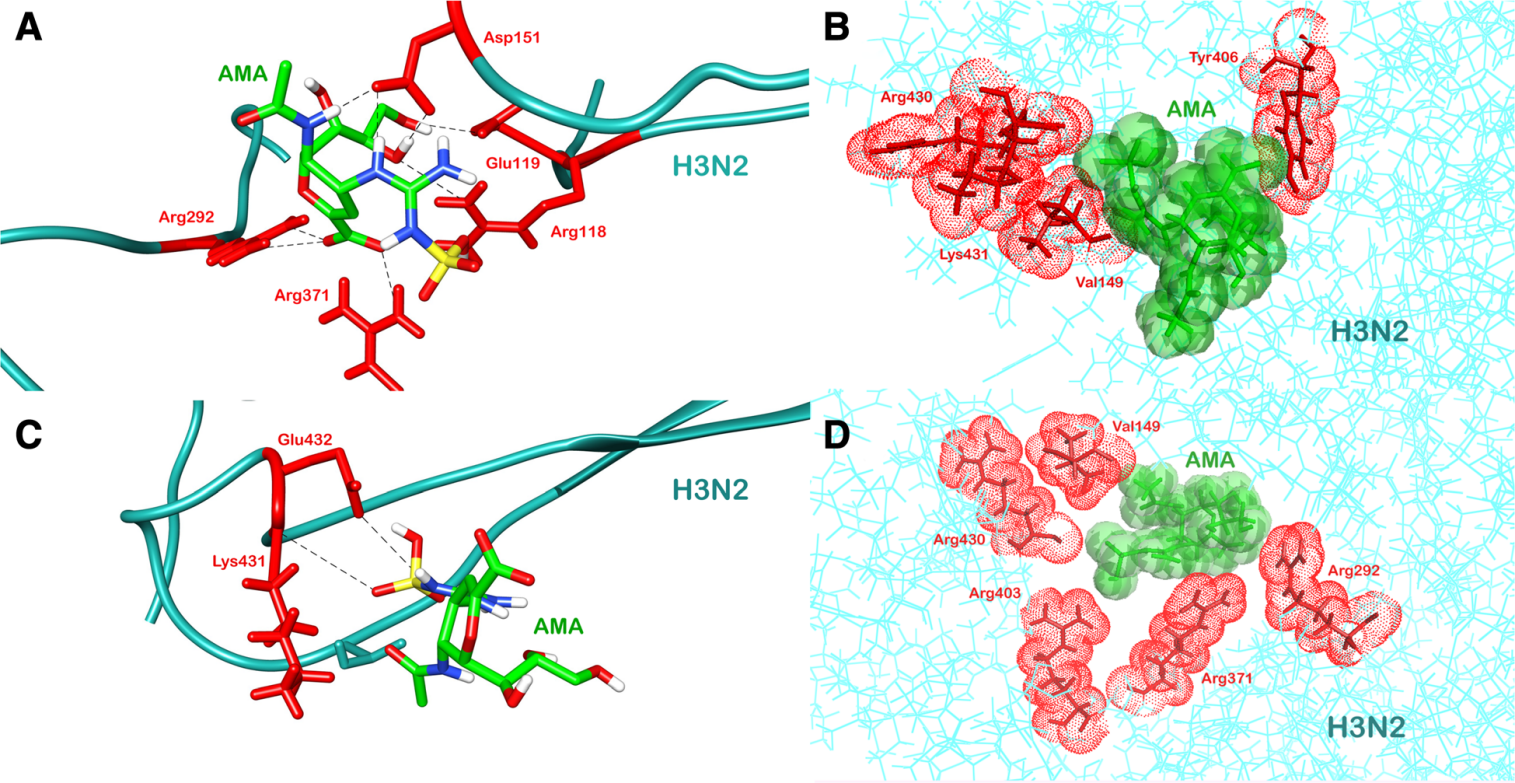
Molecular interactions of H1N1 Neuraminidase (*pink*) with AMA (*green*) depicting (**a**) hydrogen bond before MD simulations and (**b**) hydrophobic interactions before MD simulations. (**c**) Hydrogen bond after MD simulations and (**d**) hydrophobic interactions after MD simulations

**Fig. 7:**
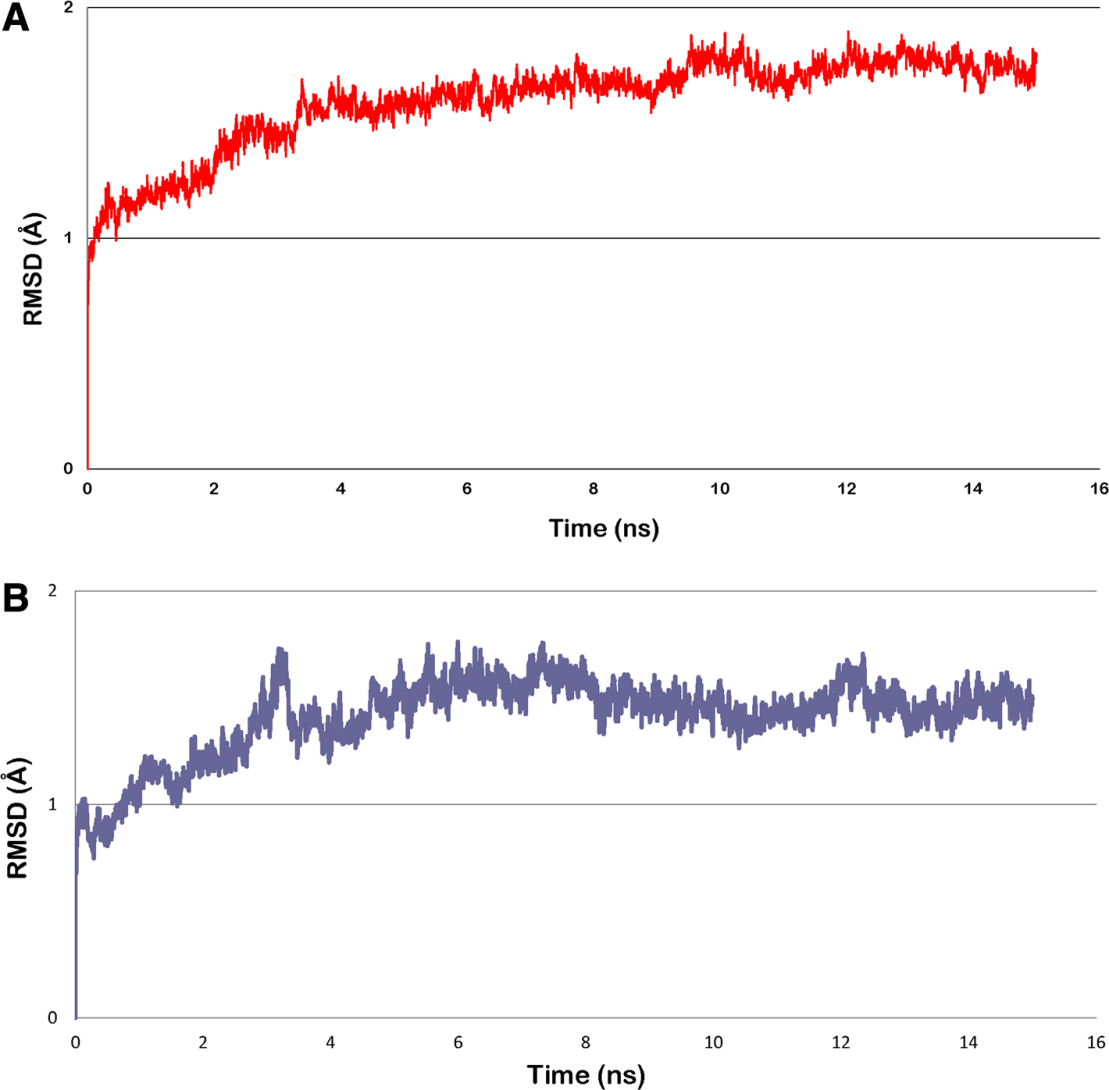
RMSD plot of molecular dynamics simulations of lead compound against NA of (**a**) H1N1 (**b**) H3N2

Docking study of the top scored compound was performed using Glide to study the interaction with crystal structures of H1N1 and H3N2. The lead compound exhibited highest predicted activity in case of both H1N1 and H3N2 models. The activity of this compound was around ten-fold higher than the next candidate as predicted by both the QSAR models. Thus this compound with highest predicted activity was selected for further analysis. The IUPAC name of the lead compound is *(2R,3R,4S)-3-acetamido-4-{[(sulfoamino)methanimidoyl]amino}f-2-[(1R,2R)-1,2,3-trihydroxypropyl]-3,4-dihydro-2H-pyran-6-carboxylic acid* (AMA). Docking of AMA with H1N1 was performed and the binding energy of the compound was found to be −8.26 Kcal/mol. Weak bonding interactions like hydrophobic and hydrogen bonds are vital parameters that stabilize interactions between ligand and protein. AMA formed various hydrogen bonds with protein residues namely Arg152, Arg156, Trp178, Glu277, Asn294, Arg371, Arg292 (Fig. 5a). It also showed hydrophobic interactions with non-polar protein residues *viz*. Glu119, Asp151, Ser 179, Arg224, Glu227 Ser246, Glu276, Asn347 and Tyr406 (Fig. 5b). Post-MD simulations AMA was found to form hydrogen bonds with residues Arg156, Asn294, Glu227, Arg371, Tyr406 (Fig. 5c) and hydrophobic interactions with Glu119, Asp 151, Agr152, Trp178 and Ser179 (Fig. 5d). Two residues of 150-loop (Asp 151 and Arg152) were observed to be interacting with AMA. Two hydrogen bonds with Glu 277 and Arg 292 were lost during simulations, however the interaction was stabilised with the ligand forming stronger hydrogen bonds. The number of hydrogen bonds between H1N1 and AMA across simulation can be seen in Additional file 1: Figure S1. The same lead compound, AMA, when docked against H3N2 showed different bonding patterns and binding energy. The compound when docked had a binding energy of −7.00 Kcal/mol. It made hydrogen bonds with Arg118, Glu119, Arg371, Asp151 and Arg292 (Fig. 6a) and hydrophobic interactions via residues Val 149, Tyr 406, Arg430, Lys431 (Fig. 6b). A difference in hydrogen bonding and hydrophobic interactions were observed post-MD simulations. AMA formed hydrogen bonds with protein residues Lys431 and Glu432 (Fig. 6c) while hydrophobic interactions with Val149, Arg292, Arg371, Arg403 and Arg430 (Fig. 6d). In this case, only one residue of 150-loop was observed to be interacting with AMA. Molecular dynamics study was performed on this lead compound and RMSD was recorded for first 15 nanoseconds to study fluctuations and conformational changes in protein which gives a measure of the stability of protein in vivo. The ligand bound protein complex of both H1N1 and H3N2 was found to be stable for a period of 11 ns and 7 ns respectively (Fig. 7). This implied that protein underwent significant structural changes during initial stages and was stable for later stage during simulation.

In order to understand the position of AMA in H1N1 and H3N2 in comparison to zanamivir, the latter inhibitor was docked and superimposed. Additional file 1: Figure S2 shows the relative position of both inhibitors in cavity. Interacting residues can be seen in Additional file 1: Figure S3. AMA in both H1N1 and H3N2 strain was observed to be binding in the cavity in a spreadout fashion by occupying and forming strong interaction with the cavity. Also, the docking score of AMA with H1N1 (−8.26 kcal/mol) and H3N2 (−7.00 kcal/mol) was observed to be better than Zanamivir with H1N1 (−6.66 kcal/mol) and H3N2 (−5.55 kcal/mol), indicating stronger interaction.

Accessible surface area (ASA) analysis of the free and docked complexes was performed by calculating the change in accessible surface area (ASA). In case of H1N1, the change in ASA was around 1411 Å^2^ while for H3N2, the change was 615 Å^2^. Though some change was observed in ASA of all residues lining the cavity of H1N1 and H3N2, four residues (Arg118, Glu119, Glu277 and Arg292) of H1N1 exhibited a significant change, indicating their importance in drug binding.

In order to understand the correlation between IC50 values and docking scores of experimentally reported dataset compounds, the two most active compounds and two least active compounds were docked and the values were compared (Additional file 1: Table S2).

## Conclusion

The objective of the present work was to gain insight into structural features of zanamivir derivatives for prediction of anti-influenza activity using GQSAR approach. This study demonstrates a correlation between structure and inhibitory activity of these molecules. Two models were generated targeting NA of both H1N1 and H3N2 influenza strains. The developed model generated various descriptors namely R1_SdOE_index, R1_6ChainCount, R1_SssSE-index, R1_SaaaCE_index, R1_SdsCHcount and R1_schiV4 in which two descriptors SssSE-index and SdsCHcount showed negative contribution while rest all showed positive contribution. A positive contribution suggests increase in contribution of that descriptor could be beneficial for inhibitory activity while a negative contribution indicates that those descriptors are detrimental for inhibitory activity. Thus, these contributions provide insights into design of novel molecule with enhanced inhibitory activity. We also developed one novel inhibitor (AMA) using the combinatorial library approach which displayed substantial binding affinity for NA in both H1N1 and H3N2 pandemic influenza strains. AMA was docked against the active site of NA and a satisfactory docking score of −8.26 Kcal/mol and −7.00 Kcal/mol was observed for H1N1and H3N2 respectively. MD simulations of AMA stabilized the ligand bound protein complex which resulted in a steady trajectory for satisfactory time. Complex structure of ligand and protein was found to be energetically stable post MD Simulations. Thus this provides evidence that the novel compound could serve as potent anti-influenza drugs with improved binding properties and low IC50 values than traditional drugs.

## Additional file

Additional file 1: Figure S1. Graph depicting number of hydrogen bonds between H1N1 and AMA across simulations. **Figure S2.** Figure comparing the conformation of AMA and Zanamivir in (a) H1N1 and (b) H3N2. **Figure S3.** Interacting residues of (a) H1N1 and (b) H3N2 with Zanamivir. **Table S1:** Structures and anti-influenza activity of acylguanidine zanamivir derivatives. **Table S2.** Table showing correlation between IC50 and docking scores of most and least active dataset compounds. (DOCX 1691 kb)

## References

[1] Nelson MI, Holmes EC (2007). The evolution of epidemic influenza. Nat Rev Genet.

[2] Neumann G, Noda T, Kawaoka Y (2009). Emergence and pandemic potential of swine-origin H1N1 influenza virus. Nature.

[3] Carrat F, Flahault A (2007). Influenza vaccine: the challenge of antigenic drift. Vaccine.

[4] Harper SA, Fukuda K, Uyeki TM, Cox NJ, Bridges CB (2005). Prevention and control of influenza, Recommendations of the Advisory Committee on Immunization Practices (ACIP). MMWR Recomm Rep.

[5] Banning M (2005). Influenza: incidence, symptoms and treatment. Br J Nurs.

[6] Skehel JJ, Wiley DC (2000). Receptor binding and membrane fusion in virus entry: the influenza hemagglutinin. Annu Rev Biochem.

[7] Wiley DC, Skehel JJ (1987). The structure and function of the hemagglutinin membrane glycoprotein of influenza virus. Annu Rev Biochem.

[8] Gong J, Xu W, Zhang J (2007). Structure and functions of influenza virus neuraminidase. Curr Med Chem.

[9] Amaro RE, Swift RV, Votapka L, Li WW, Walker RC, Bush RM (2011). Mechanism of 150-cavity formation in influenza neuraminidase. Nat Commun.

[10] Russell RJ, Haire LF, Stevens DJ, Collins PJ, Lin YP, Blackburn GM, Hay AJ, Gamblin SJ, Skehel JJ (2006). The structure of H5N1 avian influenza neuraminidase suggests new opportunities for drug design. Nature.

[11] Gubareva LV, Kaiser L, Hayden FG (2000). Influenza virus neuraminidase inhibitors. Lancet.

[12] Zambon MC (1999). Epidemiology and pathogenesis of influenza. J Antimicrob Chemother.

[13] Goyal S, Dhanjal JK, Tyagi C, Goyal M, Grover A (2014). Novel Fragment-Based QSAR Modeling and Combinatorial Design of Pyrazole‐Derived CRK3 Inhibitors as Potent Antileishmanials. Chem Biol Drug Des.

[14] Goyal S, Grover S, Dhanjal JK, Tyagi C, Goyal M, Grover A (2014). Group-based QSAR and molecular dynamics mechanistic analysis revealing the mode of action of novel piperidinone derived protein–protein inhibitors of p 53–MDM2. J Mol Graph Model.

[15] Tyagi C, Gupta A, Goyal S, Dhanjal JK, Grover A (2014). Fragment based group QSAR and molecular dynamics mechanistic studies on arylthioindole derivatives targeting the α-β interfacial site of human tubulin. BMC Genomics.

[16] Vats C, Dhanjal JK, Goyal S, Bharadvaja N, Grover A (2015). Computational design of novel flavonoid analogues as potential AChE inhibitors: analysis using group-based QSAR, molecular docking and molecular dynamics simulations. Struct Chem.

[17] Lin CH, Chang TC, Das A, Fang MY, Hung HC, Hsu KC, Yang JM, von Itzstein M, Mong KK, Hsu TA, Lin CC (2013). Synthesis of acylguanidine zanamivir derivatives as neuraminidase inhibitors and the evaluation of their bio-activities. Org Biomol Chem.

[18] Goyal M, Dhanjal JK, Goyal S, Tyagi C, Hamid R, Grover A (2014). Development of dual inhibitors against Alzheimer’s disease using fragment-based QSAR and molecular docking.

[19] Goyal S, Jamal S, Shanker A, Grover A (2015). Structural investigations of T854A mutation in EGFR and identification of novel inhibitors using structure activity relationships. BMC Genomics.

[20] Singla RK, Bhat GV (2010). QSAR model for predicting the fungicidal action of 1,2,4-triazole derivatives against Candida albicans. J Enzyme Inhib Med Chem.

[21] Golbraikh A, Tropsha A (2000). Predictive QSAR modeling based on diversity sampling of experimental datasets for the training and test set selection. Mol Divers.

[22] Afantitis A, Melagraki G, Sarimveis H, Igglessi-Markopoulou O, Kollias G (2009). A novel QSAR model for predicting the inhibition of CXCR3 receptor by 4-N-aryl-[1,4] diazepane ureas. Eur J Med Chem.

[23] Golbraikh A, Tropsha A (2002). Beware of q2!. J Mol Graph Model.

[24] Dhanjal JK, Goyal S, Sharma S, Hamid R, Grover A (2014). Mechanistic insights into mode of action of potent natural antagonists of BACE-1 for checking Alzheimer’s plaque pathology. Biochem Biophys Res Commun.

[25] Dhanjal JK, Grover S, Paruthi P, Sharma S, Grover A (2014). Mechanistic Insights into Mode of Action of a Potent Natural Antagonist of Orexin Receptor-1 by Means of High Throughput Screening and Molecular Dynamics Simulations. Comb Chem High Throughput Screen.

[26] Goyal S, Grover S, Dhanjal JK, Goyal M, Tyagi C, Chacko S, Grover A (2014). Mechanistic insights into mode of actions of novel oligopeptidase B inhibitors for combating leishmaniasis. J Mol Model.

[27] Grover S, Dhanjal JK, Goyal S, Grover A, Sundar D (2014). Computational identification of novel natural inhibitors of glucagon receptor for checking type II diabetes mellitus. BMC Bioinformatics.

[28] Halgren TA, Murphy RB, Friesner RA, Beard HS, Frye LL, Pollard WT, Banks JL (2004). Glide: a new approach for rapid, accurate docking and scoring. 2. Enrichment factors in database screening. J Med Chem.

[29] Jorgensen WL, Duffy EM (2002). Prediction of drug solubility from structure. Adv Drug Deliv Rev.

[30] Jorgensen WL, Tirado-Rives J (2005). Potential energy functions for atomic-level simulations of water and organic and biomolecular systems. Proc Natl Acad Sci U S A.

[31] Jorgensen WL, Chandrasekhar J, Madura JD, Impey RW, Klein ML (1983). Comparison of simple potential functions for simulating liquid water. J Chem Phys.

[32] Jain SK, Vishwakarma S, Nayak P. 3D QSAR analysis on pyrrolidine derivatives as DPP IV inhibitors. Int J Pharm Biomed Res. 2011;2(3).

