# Supplementary material for: Molecular modeling and lead design of substituted zanamivir derivatives as potent anti-influenza drugs
Source: BMC Bioinformatics. 2016 Dec 22;17(Suppl 19):512. doi: 10.1186/s12859-016-1374-1 (PMC5259988; doi:10.1186/s12859-016-1374-1)
Supplement: Additional file 1: Figure S1. — Graph depicting number of hydrogen bonds between H1N1 and AMA across simulations. Figure S2. Figure comparing the conformation of AMA and Zanamivir in (a) H1N1 and (b) H3N2. Figure S3. Interacting residues of (a) H1N1 and (b) H3N2 with Zanamivir. Table S1: Structures and anti-influenza activity of acylguanidine zanamivir derivatives. Table S2. Table showing correlation between IC50 and docking scores of most and least active dataset compounds. (DOCX 1691 kb) [file 12859_2016_1374_MOESM1_ESM.docx]

**Supplementary material**

**Figure S1.** Graph depicting number of hydrogen bonds between H1N1 and AMA across simulations.

**
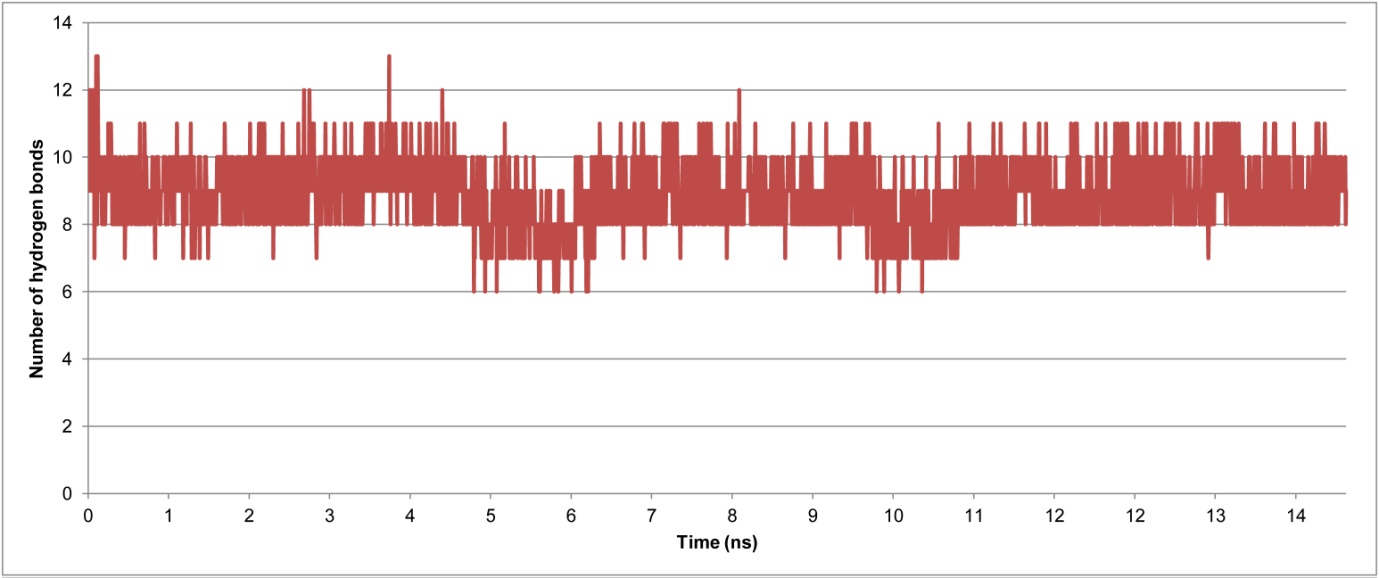
**

**Figure S2.** Figure comparing the conformation of AMA and Zanamivir in (a) H1N1 and (b) H3N2.


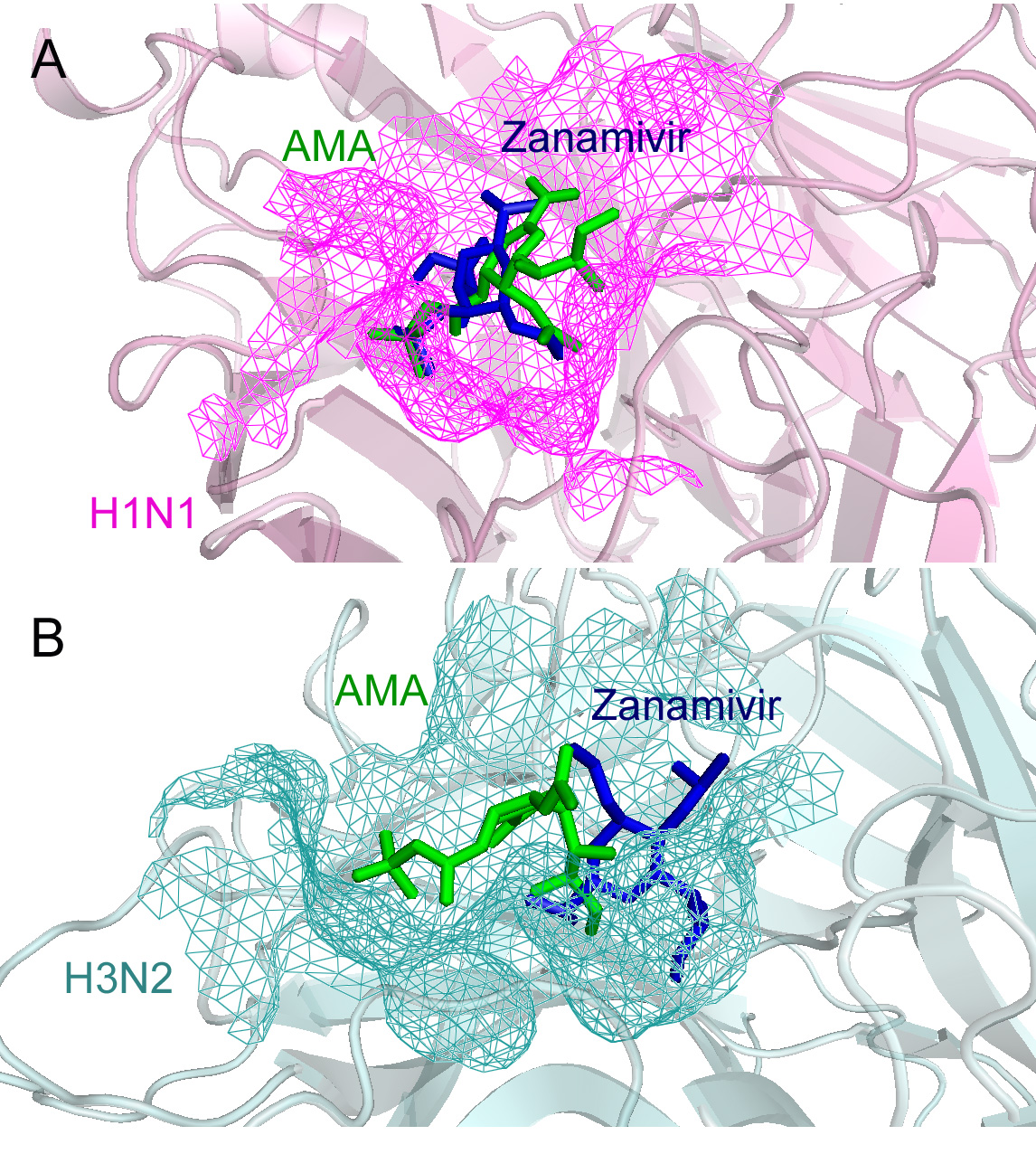
**Figure S3.** Interacting residues of (a) H1N1 and (b) H3N2 with Zanamivir.

**
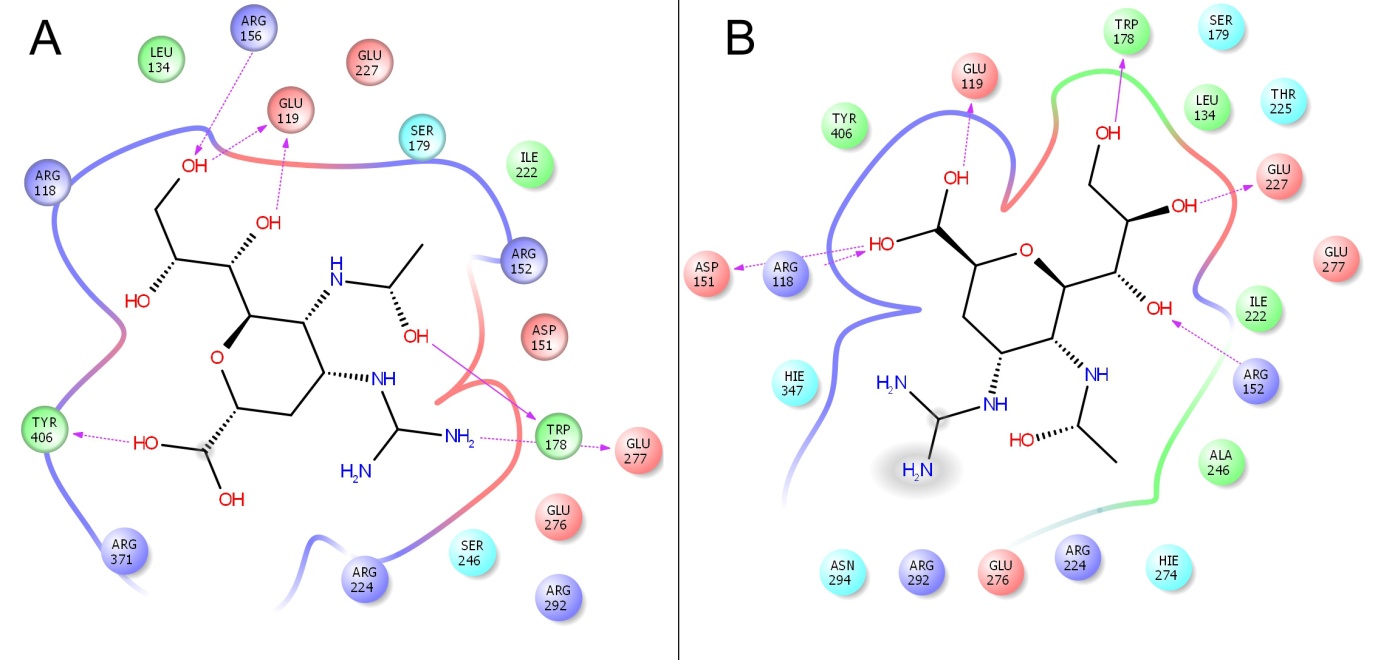
Table S1:** Structures and anti-influenza activity of acylguanidine zanamivir derivatives.

| Serial no | Compound | H1N1 | | H3N2 | |
| --- | --- | --- | --- | --- | --- |
|  |  | IC50  (nM) | pIC50 | IC50 (nM) | pIC50 |
| f | 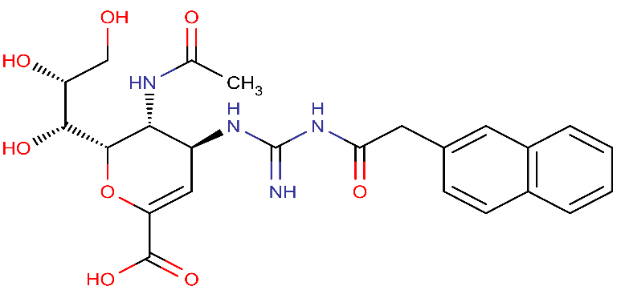 | 54.9 | -1.740 | 58.3 | -1.766 |
| g | 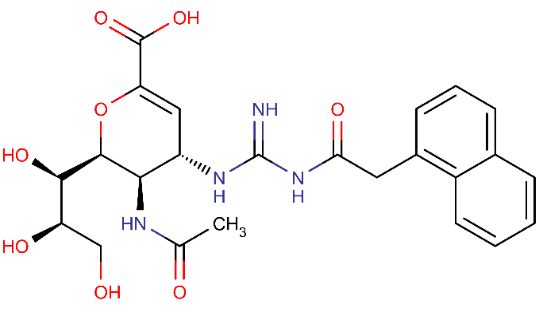 | 98.3 | -1.993 | 84.4 | -1.926 |
| h | 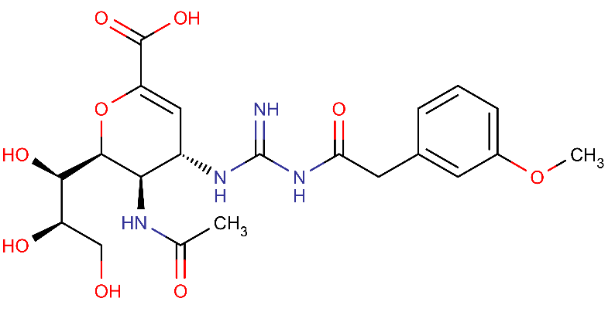 | 209 | -2.320 | 243 | -2.386 |
| i | 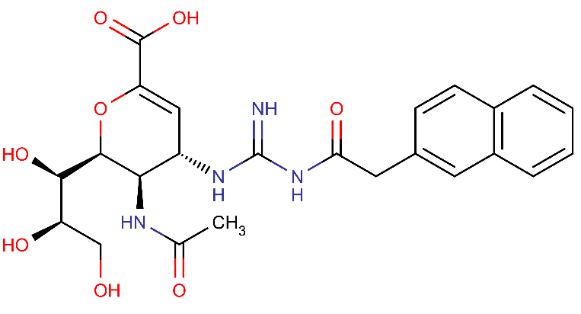 | 74.9 | -1.874 | 66.9 | -1.825 |
| j | 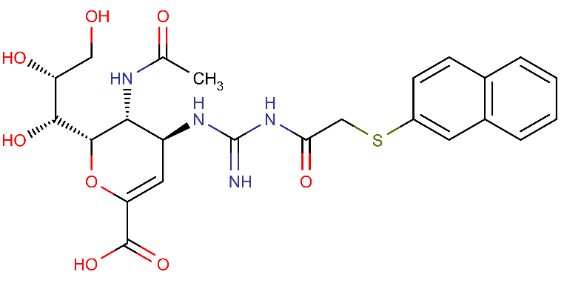 | 20.1 | -1.303 | 25.5 | -1.407 |
| l | 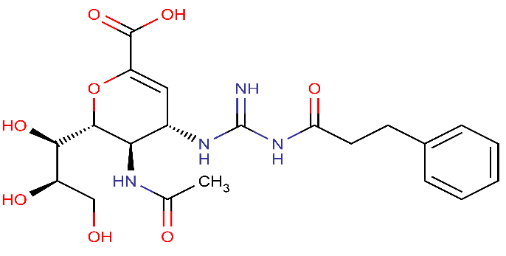 | 203 | -2.307 | 234 | -2.369 |
| m | 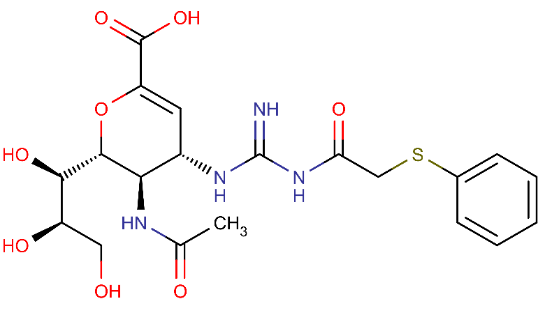 | 189 | -2.276 | 211 | -2.324 |
| n | 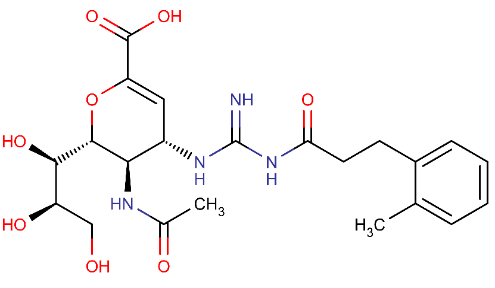 | 462 | -2.665 | 606 | -2.782 |
| o | 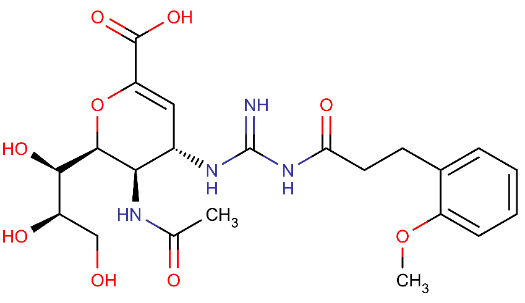 | 196 | -2.292 | 205 | -2.312 |
| q | 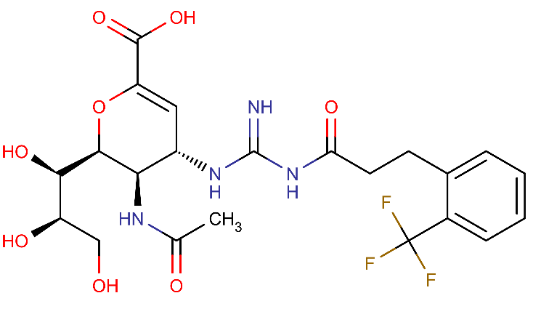 | 332 | -2.521 | 472 | -2.674 |
| r | 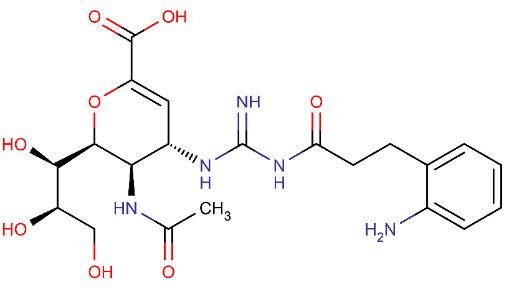 | 176 | -2.246 | 209 | -2.320 |
| s | 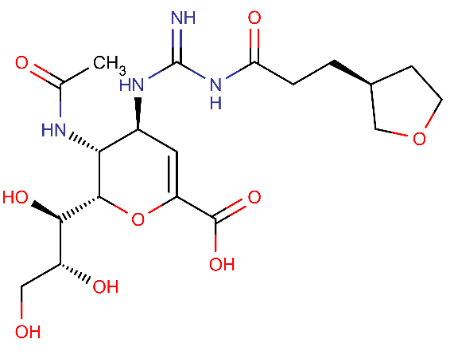 | 1200 | -3.079 | 1400 | -3.146 |
| t | 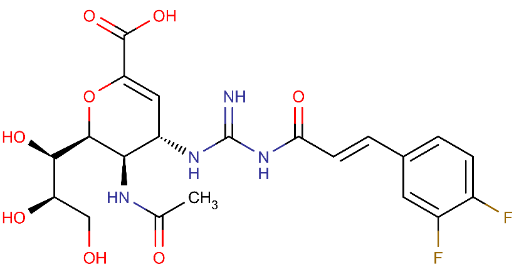 | 160 | -2.204 | 182 | -2.260 |
| u | 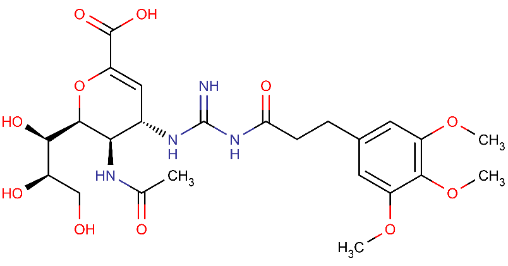 | 239 | -2.378 | 337 | -2.528 |
| v | 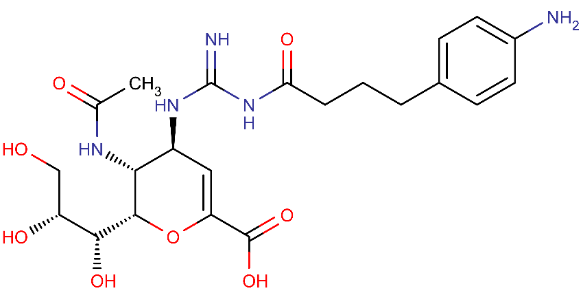 | 421 | -2.624 | 339 | -2.530 |
| w | 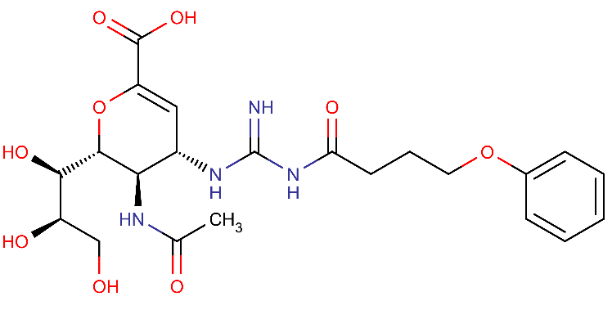 | 180 | -2.255 | 173 | -2.238 |
| x | 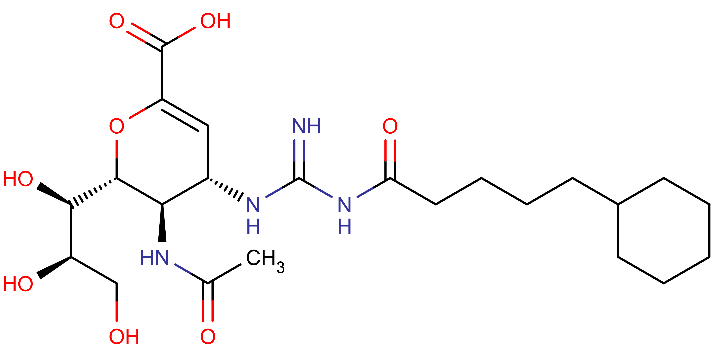 | 397 | -2.599 | 472 | -2.674 |
| y | 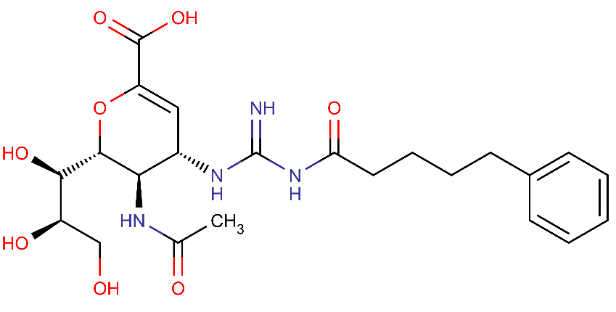 | 260 | -2.415 | 421 | -2.624 |
| z | 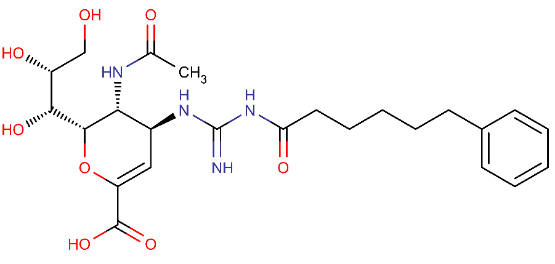 | 293 | -2.467 | 360 | -2.556 |
| aa | 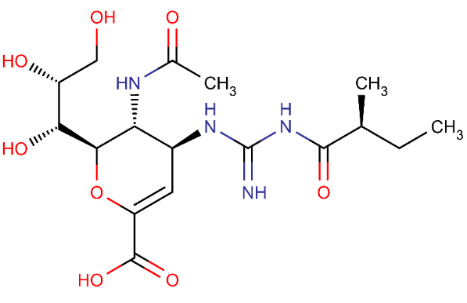 | 775 | -2.889 | 868 | -2.939 |
| ab | 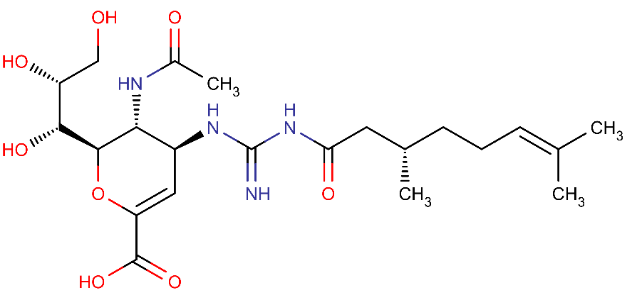 | 541 | -2.733 | 615 | -2.789 |
| ac | 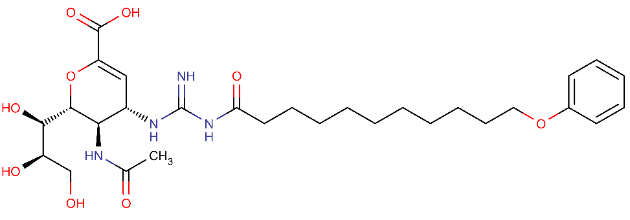 | 203 | -2.307 | 196 | -2.292 |
| ad | 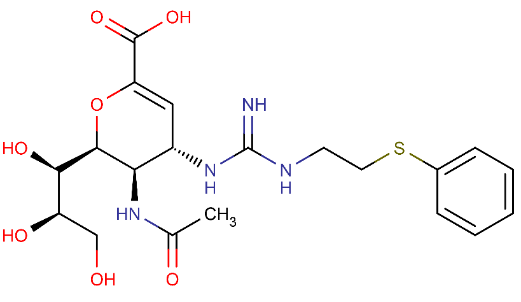 | 39400 | -4.595 | 29600 | -4.471 |
| ae | 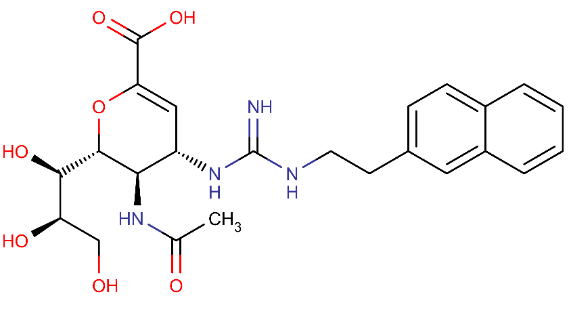 | 34644 | -4.540 | 38303 | -4.583 |

**Table S2.** Table showing correlation between IC50 and docking scores of most and least active dataset compounds.

| **S.No.** | **Compound** | **H1N1** | | **H3N2** | |
| --- | --- | --- | --- | --- | --- |
|  |  | **IC50 (nM)** | **Docking score**  **(kcal/mol)** | **IC50 (nM)** | **Docking score**  **(kcal/mol)** |
| **1** | ae | 34644 | -2.42 | 38303 | -2.20 |
| **2** | ad | 39400 | -2.38 | 29600 | -2.32 |
| **3** | f | 54.9 | -7.03 | 58.3 | -8.05 |
| **4** | j | 20.1 | -7.34 | 25.5 | -8.23 |
